# On the Origin of Rheumatoid Arthritis: The Impact of Environment and Genes—A Population Based Twin Study

**DOI:** 10.1371/journal.pone.0057304

**Published:** 2013-02-28

**Authors:** Anders J. Svendsen, Kirsten O. Kyvik, Gunnar Houen, Peter Junker, Kaare Christensen, Lene Christiansen, Christian Nielsen, Axel Skytthe, Jacob V. Hjelmborg

**Affiliations:** 1 The Danish Twin Registry, Epidemiology, Institute of Public Health, University of Southern Denmark, Odense, Denmark; 2 Institute of Regional Health Research, University of Southern Denmark and Odense Patient data Explorative Network (OPEN), Odense University Hospital, Odense, Denmark; 3 Department of Clinical Biochemistry and Immunology, Statens Serum Institute, Copenhagen, Denmark; 4 Department of Rheumatology, Odense University Hospital, University of Southern Denmark, Odense, Denmark; 5 Department of Clinical Immunology, Odense University Hospital, Odense, Denmark; 6 Epidemiology and Statistics, Institute of Public Health, University of Southern Denmark, Odense, Denmark; South Texas Veterans Health Care System and University Health Science Center San Antonio, United States of America

## Abstract

**Background:**

Rheumatoid arthritis (RA) is an autoimmune disease with a complex origin. Previous studies have reported heritability estimates on RA at about 60%. Only 16% of the genetic background of the disease has been disclosed so far. The purpose of the present investigation was to provide an optimized estimate on the heritability of RA and to study the recurrence risk in a nationwide Caucasian twin population.

**Methods and Findings:**

In a mail survey addressed to 56.707 twin individuals, RA was reported by 479 individuals, mean age 52 (range 16–73). Respondents underwent an interview and clinical examination. Ascertainment probability was 80%. RA was confirmed in 162 twin individuals yielding a prevalence at 0.37% (95% CI 0.31–0.43). The mean discordance time was 19 years (range 0–57). The concordance was 9.1% (95% CI 1.9 to 24.3) in MZ, 6.4% (95% CI 2.1 to 14.3) in DZss. The increased relative risk of attracting RA conditioned on having an affected cotwin compared to the background population risk was 24.6 to 35.4 in MZ twins and 17.3 to 31.6 in DZss twins. The correlation coefficients were 0.60 (0.33 to 0.78) in monozygotic (MZ) and 0.55 (0.33 to 0.72) in dizygotic same sexed (DZss) pairs. Twelve percent (95% CI 0–76%) of the phenotypic variance in the liability to RA was due to additive genetic effects, 50% (95% CI 0–72%) to shared environmental effects and 38% (95% CI 17–61%) to non-shared environmental effects.

**Conclusions:**

This study emphasizes that family factors are important for the development of RA. Although genetic effectors are important, shared and non-shared environmental triggers and/or epigenetic stochastic events seem to be even more significant. However, it should be borne in mind that the genetic and non-genetic components may not be the same across disease subsets.

## Introduction

Rheumatoid arthritis (RA) is a chronic systemic disorder with autoimmune traits in which polyarticular synovitis is particularly prominent. [1] Its origin remains elusive but there is evidence, that both genetic and environmental triggers are implicated in the pathogenesis and growing evidence suggests that RA consists of at least 2 different subsets characterized by the presence or absence of antibodies to citrullinated protein antigen (ACPA). [2] Newly published genome-wide association studies (GWAS) have demonstrated several DNA sequence variations associated with RA. [3] In a recent GWA metaanalysis it was estimated that current genetic discoveries account for around 16% of the disease variance, [4] although more than half of the liability to RA was considered to be genetic, [5] regardless of autoantibody status. [6] The most cited heritability estimate at around 60% is derived from 2 previous twin studies. The population based Finnish Study by Aho et al. was based on record linkage between the Finnish Twin Register and the Sickness Insurance Register but without diagnostic validation. [7] The study by McGregor et al. from the UK was based on volunteer RA subjects recruited from various sources, thus implying a risk of selection bias. [8] The probandwise and the pairwise concordance estimate in the Finnish study was 22.0% and 12.3% in monozygotic (MZ) twins and 6.7% and 3.5% in dizygotic same sexed (DZss) twins. The pairwise concordance estimate was 15.4% in MZ twins and 3.6% in same- and opposite sexed dizygotic (DZss/os) twins in the UK study.

In 2002 we published a probandwise concordance estimate at 0% in MZ and 8.8% in DZss/os twins suggesting a significant role for non-genetic effector mechanisms in the causation of RA. [9] This study was based on a mail survey in 1994 to unselected twin birth cohorts including the decades1921 to 1930 and 1953 to 1982. Meanwhile, The Danish Twin Register has been expanded to include also the 1931–1952 cohorts. [10] Our purpose was, based on this large and ethnically homogenous population of RA twins, to provide an optimized heritability estimate on RA and to study the recurrence risk of the disease.

## Methods

### Subjects

Twin individuals with RA were identified from the nationwide population based twin cohorts born 1921 through 1982. [11] A questionnaire asking whether they had ever been diagnosed with rheumatoid arthritis was addressed to all available twins born 1921–1930 and 1953–1982 in 1994 and all available twins born 1931–1982 in 2002. Thus, twins born 1953–1982 were addressed twice. Individuals reporting RA in 1994 and/or in 2002 were subsequently contacted by mail and phone in 1995 and in 2008 respectively. Subjects in whom RA could not be ruled out by telephone interview and/or information from physicians were invited to participate in an in-person structured interview and clinical examination both in 1994 and 2002. The twins were visited at home by the principal investigator (AJS) or a research nurse and a standardized joint examination was undertaken. Available medical records were collected. The diagnosis was confirmed according to the modified ARA 87 criteria which takes into account criteria fulfilled currently as well as in the past. [12].

### Zygosity

Zygosity determination on same-sexed twins in the Danish Twin Registry was based on the questionnaire method which has been proved to assign correct zygosity in 95% of all twin pairs compared to zygosity determined by genetic markers. [13] In addition, in same-sexed RA twin pairs, zygosity was also determined by genetic markers.

### Autoantibodies

Anti-cyclic citrullinated peptide antibodies (ACPA) were determined by ELISA (Euro-Diagnostica, Malmö, Sweden) as described by the manufacturer.

IgM antibodies against IgG (IgM-RF) were determined by ELISA using purified IgG as antigen and peroxidase-conjugated F(ab)2 rabbit immunoglobulin (DAKO, Copenhagen, Denmark).

### HLA Typing

HLA-DRB1 genotyping was performed using LABType SSO DRB1 Typing Test kit (One Lambda), coupled with the Luminex xMAP technology (Luminex) according to the manufacturer’s instructions.

### Record Linkage

The Danish National Patient Registry (NPR) covers all inpatients in Danish hospitals since 1977 and in addition outpatients since 1995. [14] We used record linkage with NPR using the unique personal identification number assigned to all persons with a permanent residence in Denmark to find twins with RA who had not been identified in the surveys. In the 1994 survey cases solely identified in NPR were validated on an individual basis by retrieval of medical records. On the basis of the capture-recapture model [15] our 1994 survey had an estimated probability of ascertainment of 78.3%. [9].

In the 2002 survey cases solely identified in NPR were validated by a frequency approach. The validity of RA diagnoses in NPR has previously been investigated according to the ACR 1987 criteria [16]. The overall confirmation rate was 46% but dependent on the type of department and the number of hospital registrations. Twins identified in NPR were categorized according to the number of registrations with RA and whether they had been discharged from a rheumatology department or other department. The number in each category was then multiplied with the corresponding confirmation rate to reach the expected number with true RA. The completeness of ascertainment of the 2002 survey was also estimated by the capture-recapture method. [15].

The ascertainment was further scrutinized by record linkage with the Danish Register of Causes of Death. Classification of cause(s) of deaths has been done according to WHO’s rules and since 1994 by ICD-10 codes. [17].

### Statistical Analysis

A proband was an RA twin who independently reported RA and a secondary case was a twin ascertained through the cotwin.

The study comprised three categories of RA twin pairs. Singly ascertained concordant pairs–that is, one affected twin was a proband and the other a secondary case (C1); doubly ascertained concordant pairs–that is, both affected twins were probands (C2). Among discordant pairs the affected twin fulfilled the proband criteria (D1).

The casewise concordance is defined as (2C1+2C2)/(2C1+2C2+D1), the probandwise as (C1+2C2)/(C1+2C2+D1) and the pairwise as (C1+C2)/(C1+C2+D1). [18] Both the casewise and the pairwise estimates vary with the ascertainment probability and are therefore not comparable between studies unless the studies achieve identical levels of ascertainment. The probandwise concordance can be considered an estimate of the casewise concordance that is robust to incomplete ascertainment. In case of complete ascertainment the casewise and the probandwise rates are identical. Furthermore, the probandwise concordance can be directly compared to the recurrence risk in other sets of relatives. The probandwise concordance rate divided by the population prevalence will give the increased risk of RA conditioned on having an affected cotwin compared to the risk in the background population.

Since some twin pairs in our study became concordant between the mail surveys and the clinical examination not all twins from concordant pairs had the opportunity to report RA. Therefore, the probandwise concordance rate in our study is likely underestimated and the casewise rates can also be regarded as a sensitivity analysis of the probandwise concordance rate given that all the secondary cases had been probands. The casewise concordance rate was adjusted for sex.

In addition to the concordance rates we also present the tetrachoric correlation (the correlation of liability) [19] which is the polychoric correlation of binary traits and also takes into account twin pairs where neither twin is affected with RA. This statistic assumes that, underlying the observed division of twins into those with and without RA, there exists a latent vulnerability or liability to RA. It is assumed that a threshold exists on this liability scale such that individuals with a liability above the threshold develop RA while those below the threshold remain free of RA. The population prevalence reflects this threshold in the general population. The tetrachoric correlation represents the correlation in twins for this underlying liability to RA. It is further assumed that RA has a multifactorial etiology involving a number of genetic and environmental risk factors of small to moderate effect such that the liability to RA in the general population will be approximately normal. We have estimated the twin population prevalence by dividing the number of twins with RA with the number of responders and age- and sex adjusted to the 2004 Danish population. An estimate of the increased relative risk of attracting RA conditioned on the having an affected cotwin compared to the background population risk, was calculated by dividing the casewise and probandwise concordance rates respectively with the twin population prevalence.

Heritability is a dimensionless population parameter that allows a comparison of the relative importance of genes and environment to the variation of traits within and across populations. [20] The proportion of variance in liability due to additive genetic effects is termed the heritability and was estimated by the method of analysis based on structural equation models. [21] A polygenic, multifactorial liability model was fitted to the data. The calculation is based on the tetrachoric correlations. The following components of variance were tested in the models: the additive genetic effect (A) measures the fraction of variation between individuals in a population that is due to their genotype, the dominance genetic effects (D) measures the variation due to interactions between alleles at the same locus, the shared environmental effects (C) measures the variation between twin pairs and unique environmental effects (E) the variation within twin pairs. Models containing D and C cannot be tested simultaneously. The components of variance were adjusted for sex.

The model with the lowest value of Akaike’s information criterion (AIC) reflects the best balance between goodness of fit and parsimony. [22].

All estimates are given with 95% confidence intervals.

The study was conducted and reported in accordance with the Strengthening and the Reporting of Observational Studies in Epidemiology (STROBE) recommendations [23].

### Ethics

The study was approved by all the regional scientific ethics committees in Denmark (Projekt ID: S-20070088) and the Danish Data Protection board (J.nr. 2007-41-0747). We obtained informed written consent from all participants in the study.

## Results

A total of 56,707 twin individuals were enrolled of which 45,280 responded yielding a response rate at 80%. In the 1994 and the 2002 surveys the response rates were 81.4% and 75% respectively. The responders represented 27,671 twin pairs. The response rate was higher among women (82%) than men (74%). Concordant responses were more frequent in MZ twin pairs (70%) than among DZ same sex pairs (60%) and DZ opposite sex pairs (54%). The final study base comprised 26,470 twin pairs of which 16,852 (63.7%) were intact pairs. Subsequently, when the 479 twins reporting RA were traced, 42 (9%) were non-responders to follow-up, had secret addresses and telephone numbers, 42 (9%) had died, 4 (8 ‰) had emigrated. Among the twins traced, 223 (47%) did not have RA based on telephone interview and/or medical records. Osteoarthritis (19%) was the most prevalent cause of exclusion. Among the remaining twins 97% accepted to participate in the clinical examination and a total of 173 (incl. 5 secondary cases) fulfilled the modified ARA 87 criteria [12] of which 11 had competing diagnoses and were excluded. Table 1 presents the characteristics of the remaining 162 RA twins. There were no significant differences between MZ and DZ twins in any of the listed variables.

**Table 1:** Demographic and clinical data on twins with rheumatoid arthritis according to zygosity.

Characteristic	MZ (n = 34)		DZss (n = 81)		DZos (n = 47)		MZ DZss difference (95% CI)	
Women	28	82.4	56	69.1	37	78.7	−13.2	(−29.5 to 0.03)
Ever positive for rheumatoid factor	28	82.4	64	80.0	38	80.9	2.4	(−13.2 to 17.9)
Ever positivefor anti-CCP	24	70.6	63	78.8	38	809	−8.2	(−25.9 to 9.9)
Nodules ever	20	58.8	36	44.4	20	42.6	14.4	(−5.4 to 34.1)
Erosions ever	21	61.8	57	70.4	34	72.3	−8.6	(−27.8 to 10.5)
Positive forshared epitope	23	67.7	65	81.3	36	76.6	−13.6	(−31.5 to 4.3)
Homozygote for shared epitope	14	41.2	29	36.3	12	25.5	4.9	(−14.7 to 24.5)
Mean (SD) age (years)	54.5	2.1	58.7	1.3	58.0	1.37	−4.15	(−9.0 to 0.7)
Mean (SD) age at onset (years)	39.2	2.5	44.5	1.5	44.3	1.6	−5.2	(−10.9 to 0.3)
Mean (SD) discordance time (years)	20.1	2.1	18.4	1.3	20.3	1.8	1.8	(−2.9 to 6.5)

Figures are number (percentage) of individuals unless otherwise stated.

MZ denotes monozygotic, DZss dizygotic same sexed, and DZos dizygotic opposite sexed.

**Figure 1:**
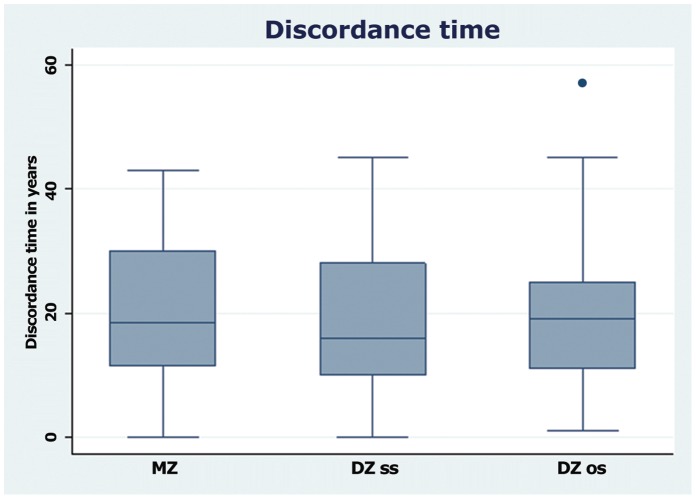
Discordance time (years) in RA affected twin pairs according to zygosity. MZ denotes monozygotic, DZss dizygotic same sexed, and DZos dizygotic opposite sexed. From the bottom up the smallest observation, lower, median, upper quartile, and the largest observation is shown. There is one outlier among the DZos pairs.

The box plot shows the distribution of discordance time in each zygosity group (Fig. 1). There was no difference in mean discordance time between any of the zygosity groups. The point prevalence of RA was 0.20% (95% CI 0.14% –0.27%) in men, 0.54% (95% CI 0.44% –0.64%) in women and 0.37% (95% CI 0.31% –0.43%) in men and women, age- and sex adjusted to the 2004 Danish population. The increased relative risk of attracting RA conditioned on having an affected cotwin compared to the background population risk was 24.6–35.4 in MZ twins and 17.3–31.6 in DZss twins.

### Record Linkage

The record linkage identified 86 twins with RA of which 69 had also been identified in the 2002 survey, thereby reaching an ascertainment probability at 80.2%. Two additional possible DZ concordant pairs were identified and one twin from each of these pairs reported RA but did not want to participate in the clinical examination. One further MZ pair was concordant. This twin pair was not eligible in the 1994 survey, one twin had deceased before the 2002 survey and the other did not respond.

Record linkage between twins born 1920 to 1982 and the Danish Register of Causes of Death, identified 10 twins where RA was stated in the death certificates. According this registry none of these pairs were concordant for RA.

Table 2 presents the concordances rates by zygosity and ACPA status. Adjustment for sex had only minor effects on the estimates. There was no significant difference in any of the concordance rates between MZ and DZss or between DZss and DZos. The probandwise concordance estimate was 9.1% in MZ twins, 6.4% in DZ same-sexed twins and 2.2% in opposite-sexed twins. All the seven RA concordant twin pairs were also concordant for the shared epitope except one DZss pair. Restricting the analysis to ACPA positive RA cases tended to increase the risk in MZ twins more than in DZss twins.

**Table 2:** Tetrachoric correlations and concordance rates of rheumatoid arthritis in Danish twins according to zygosity and ACPA status.

All RA twins	Zygosity	No. of ConcordantPairs	No. of Discordant Pairs	TetrachoricCorrelations	Concordance rate %					
					$Casewise (95% CI)		*Probandwise (95% CI)		Pairwise(95% CI)	
	MZ	2	30	0.60 (0.33 to 0.78)	13.1	(3.7 to 45.9)	9.1	(1.9 to 24.3)	6.3	(0.8 to 20.8)
	DZss	4	73	0.55 (0.33 to 0.72)	11.7	(4.8 to 28.7)	6.4	(2.1 to 14.3)	5.2	(1.4 to 12.8)
	DZos	1	45	0.56 (0.08 to 0.83)	4.3	(0.5 to 14.5)	2.2	(0.1 to 11.7)	2.2	(0.1 to 11.5)
Only ACPA positive RA twins										
	MZ	2	19	0.68 (0.40 to 0.84)	18.7	(5.7 to 62.0)	13.6	(2.9 to 34.9)	9.5	(1.2 to 30.4)
	DZss	4	55	0.64 (0.40 to 0.79)	14.9	(6.2 to 35.7))	8.6	(2.9 to 19.0)	6.8	(1.9 to 16.5)
	DZos	1	36	0.59 (0.11 to 0.85)	5.3	(0.6 to 17.8)	2.7	(0.1 to 14.2)	2.7	(0.1 to 14.2)

Values in parenthesis are 95% confidence intervals. ACPA denotes anti-citrullinated peptides antibody-positive rheumatoid arthritis. MZ denotes monozygotic, DZss dizygotic same sexed, and DZos dizygotic opposite sexed.

* There was one secondary case among the MZ concordant pairs, three among the DZ concordant pairs and one among the DZos pairs.

$ The estimates of the casewise concordance of MZ and DZss twins are adjusted for sex.

### Heritability

The full models, ACE (AIC −45598.912) and ADE (AIC −45595.242) were comparable with respect to Akaikés criterion. There was no significant deterioration in fit when A was dropped from the ADE model (p = 0.740, AIC −1.890) whereas reducing an ACE to a CE model resulted in a borderline significant deterioration in fit (p = 0.055, AIC 1.669). Despite the fact that A could be dropped from a statistical viewpoint, we included it in the model because there is ample evidence that genetic risk factors exist e.g. the shared epitope and PTPN22. [24] Since the tetrachoric correlations were very close between MZ pairs (0.62 (95% CI 0.27–0.83)) and DZss pairs (0.56 (95% CI 0.48–0.64)), common environment seems to contribute to RA susceptibility. Thus, the ACE model was selected as representing the best balance between fit and parsimony. The estimates from this sex adjusted model suggest that 12% (95% CI 0–0.76%) of the phenotypic variance in the liability to RA is due to additive genetic effects, 50% (95% CI 0–72%) to common environmental effects and 38% (95% CI 17–61%) to unique environmental effects.

Statistical packages: Neale MC. Mx statistical Modeling. http://openmx.psyc.virginia.edu/openmx-features.2010. Stata, version 10.

## Discussion

This study shows, that there is a considerable familial aggregation of RA as reflected by recurrence risks at 9,5–13,1 in MZ co-twins and at 6,4–11,7 in DZss co-twins as opposed to a background population risk at only 0,37%. Furthermore, since the majority of MZ twins are discordant for RA, potential triggers in the environment should be considered emphasized by the small differences in concordance and tetrachoric correlations between MZ and DZss twin pairs. In addition, our estimate of RA heritability only amounts to 12% while shared and non-shared environmental effects account for 50 and 38% respectively. By restricting the analysis to ACPA positive RA, we observed only a marginal increase in the difference of RA concordance and correlation between MZ and DZss.

Our estimates are somewhat at variance with the most cited figures on the genetic contribution to RA based on twin studies. [5], [7], [8] The UK study was based on recruitment of twins from rheumatologists and through nationwide multimedia campaigns. This implies that it was not possible to calculate prevalence or ascertainment probability. As pointed out previously, and confirmed in this study, there is a risk of over ascertainment of female twins, MZ twins and concordant pairs. [25], [26] The risk of selection bias of MZ concordant pairs in the UK study is emphasized by the fact that the number of concordant pairs was smaller than the total number expected to be concordant just by coincidence in the UK, particularly regarding DZ pairs. [9].

The Finnish population based study was a record linkage study of the Finnish Twin Cohort and the Sickness Insurance Register. The diagnosis relied on documents from the Insurance Register submitted by the attending physician, [7] and the twins were not approached and examined. Thus, no clinical characteristics were presented to compare the zygosity groups or the representativeness of RA twins and the diagnosis was not verified according to classification criteria. Besides, there was a risk of inflated concordance rates because the eligibility for free drugs could be proposed and approved on a less stringent basis, if, for example, the fact that the cotwin had RA was used as a criterion in support of the diagnosis.

The disparities between previously published recurrence risk ratios of RA among first degree relatives probably reflect differences in ascertainment probability, epidemiologic measures of occurrence, clinical characteristics and follow-up time. [27] By contrast, in population based studies like the present, it is in the order of 0.9 to 2.4 [28] and among siblings down to 1.1 [29] In a recent Swedish register linkage study the sibling recurrence risk was up to 4.6 whereas the twin recurrence risk in a combination of both MZ and same sexed DZ twins was 6.5 and thus in accordance with our estimate. [30] Taken together, these figures indicate that the recurrence risk in twins is higher than in ordinary siblings. By contrast, the recurrence risk in spouses is low, zero to 1.2, [29], [30] and therefore does not support that shared environment in late life is involved in RA. Since our data suggest that the majority of environmental impact is attributable to shared factors, it indicates that exposures in early life may contribute to RA development. Birth weight [31], smoking [32] and maternal MHC gene composition [33] are examples of risk factors in utero; breast-feeding in perinatal life [34] and exposure to pets are risk factors in the prepubertal period of life. [35] There is only week evidence to suggest an effect of early life infections. Infection during the first year of life only showed a borderline risk of RA in later life and Maternal infections during pregnancy were unrelated to the risk of RA. [36].

Recently, the impact of epigenetic changes on expression of silencing genes has attracted considerable attention. Thus, growing evidence from animal and human studies suggests, that stochastic epigenetic alterations - rather than the non-shared environment - may account for the discordance between monozygotic twins. [37]–[39] The potential involvement of stochastic events reduces the possibility to elucidate potential environmental triggers and will tend to diminish our estimate of unique environmental effectors.

There is recent evidence that ACPAs are more closely associated with the shared epitope than with RA itself [40] and that smoking, the presently most well documented environmental risk factor, is only associated with ACPA positive RA, [41], [42] emphasizing a strong gene-environment interaction in this disease subset. The present data, and our recent reports on ACPA in twins, suggest a higher genetic contribution in the ACPA positive vs. the ACPA negative RA subset and that there exist a genetic contribution to the production of ACPA beyond the shared epitope and the PTPN-22 polymorphism. [43], [44] A reevaluation of the UK twin data presented almost identical heritability estimates between ACPA positive and ACPA negative RA. [6].

Although we have involved a large cohort of twins in our surveys the estimate of heritability based on structural equation modeling is hampered by imprecision due to the binary phenotype of RA, the low prevalence and the low concordance rates. This has been demonstrated in simulation experiments testing the statistical power of the classical twin studies to resolve sources of familial resemblance of binary outcomes. [45] However, the relatively high recurrence risk among cotwins compared to background population risk and the close recurrence risks between MZ and DZss cotwins, indicate that shared environment is etiologically important. Based on the already established association between RA and its two major genetic risk factors, HLA and PTPN22 polymorphisms in Caucasians, [24] a genetic component should be considered in the model. Provided that the recurrence risk in DZ twins is higher than in ordinary siblings it seems that long term effects of early shared environment are important. This component has not been taken into account in previous heritability estimates on RA. [5], [6] Reducing a full ACE model to an AE model, thereby neglecting the effect of shared environment, will inevitably increase the additive genetic component as well as the precision of the remaining components. This may contribute to the previously reported much higher heritability estimates at 60%.

The twins participating in the current study constitute an unselected sample of birth cohorts enrolled in the Danish Twin Registry. It has previously been demonstrated that Danish twins have increased mortality during the first year of life due to prematurity, but that mortality and health trajectories in adulthood are similar to singletons. However, it is well documented that severe RA is associated with excess mortality [46]–[48] and that severity is associated with genetic susceptibility. [49]–[52] Thus, in this historical cohort study there might be a selection bias towards twins with milder disease and loss to follow up. But the clinical characteristics of our RA twins seem to be comparable to RA cases recruited from clinical settings with regard to extraarticular manifestations and auto-antibody profile and we have probably underestimated erosive disease since we did not require an updated X-ray status. The record linkage with the Danish Register of Causes of Death did not reveal any additional concordant pairs.

Generally, twin studies should report probandwise concordance. [18] Nonetheless, our estimate on the probandwise concordance rate is probably too low because all the secondary cases might have been registered as probands since they developed RA after the postal survey but before clinical examination. The casewise concordance rates in our study may therefore represent a more just estimate of the recurrence risk in twins.

There were 36.3% incomplete participating pairs who did not have the opportunity to be doubly ascertained. However, none of the RA affected twins in these pairs reported RA on behalf of the cotwin, and none of the cotwins were identified in the Danish National Patient Registry. As the proportion of intact pairs was higher for MZ than DZ twins, any inherent bias of this kind would lead to an overestimate of MZ concordance compared to DZ concordance.

The estimated ascertainment probability of RA among the responders was 80%. Since we only found one concordant MZ and two concordant DZ pairs among the RA non-reporters in the Danish National Patient Registry and none in the Danish national registry of deaths, there is no reason to believe that complete ascertainment would seriously distort the observed proportion of concordant pairs.

Cross sectional studies do not reflect the lifetime risk of RA for cotwins. However, this may not imply a significant source of bias, provided that the mean and distribution of discordance time do not differ between MZ and DZ twins. The mean discordance time in our study amounted to almost 20 years in all zygosity groups and with a comparable distribution between MZ and DZ twin pairs.

There is a higher response rate among MZ than DZ twins in volunteer based twin studies [25] and we have therefore paid major attention to acquire high and equal ascertainment probabilities between the zygosity groups. Thus, all twins invited to participate were offered a visit at home or at work. This option probably contributes to the 97% participation rate in the clinical examination. As our study shows MZ twins are more likely to be concordant for response than DZ twins, which may lead to selection bias towards overrepresentation of MZ concordant pairs. It is unlikely that the low concordance rates were due to observer bias as none of the healthy cotwins showed any signs of arthritis and disease classification was supported by medical records and blood samples. Finally, there is growing evidence for shared genetic loci in separate autoimmune diseases including RA, insulin dependent diabetes mellitus, celiac disease and inflammatory bowel disease. [53]–[56] Unfortunately, there is no validated information on other autoimmune diseases in our sample of RA affected twin pairs.

The study base consisted of unselected twin birth cohorts which enabled us to calculate an RA prevalence at 0.37%. The most recent estimate of the prevalence in Danish singletons was 0.35. [57] Both studies had an estimated ascertainment probability of 80%, used the same classification criteria and both populations consisted entirely of Danish Caucasians. Furthermore, both estimates were age and sex adjusted to the 1994 Danish population. These almost identical prevalence estimates in twins and singletons based on homogenous populations of Caucasian origin support the external validity of our study.

In conclusion this study emphasizes that family factors are important for the development of RA. Although genetic effectors are important, shared and non-shared environmental triggers and/or epigenetic stochastic events seem to be even more significant. However, it should be borne in mind, that the genetic and non-genetic components may not be the same across disease subsets.
